# Supplementary material for: Modulating adsorbed hydrogen drives electrochemical CO2-to-C2 products
Source: Nat Commun. 2023 Aug 1;14:4615. doi: 10.1038/s41467-023-40412-9 (PMC10394046; doi:10.1038/s41467-023-40412-9)
Supplement: Supplementary file 1 — Supplementary Information [file 41467_2023_40412_MOESM1_ESM.pdf]

Supplementary Information for

# Modulating Adsorbed Hydrogen Drives Electrochemical $CO_2$-to-$C_2$ Products

Jiaqi Feng, Libing Zhang, Shoujie Liu, Liang Xu, Xiaodong Ma, Xingxing Tan, Limin Wu, Qingli Qian, Tianbin Wu, Jianling Zhang, Xiaofu Sun*, Buxing Han*

## Supplementary Figures and Tables

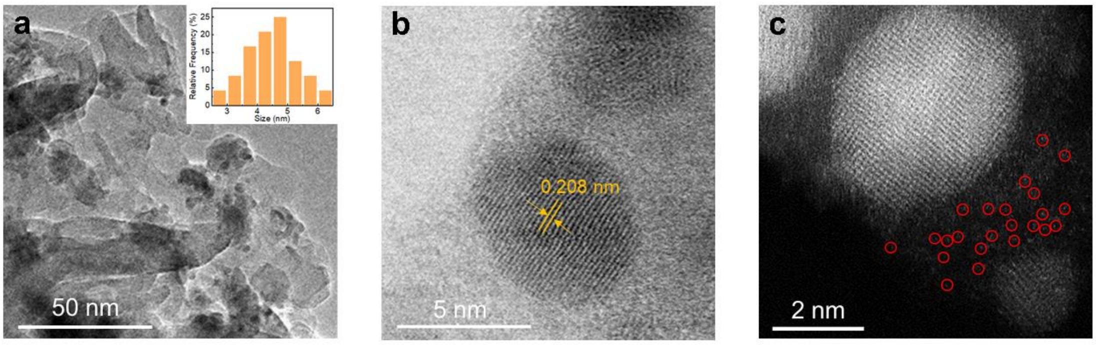


**Figure S1.** (a) TEM image (inset shows particle size distribution), (b) HRTEM image and (c) aberration-corrected HAADF-STEM image of P-$Cu_1$/$Cu_{NP}$.

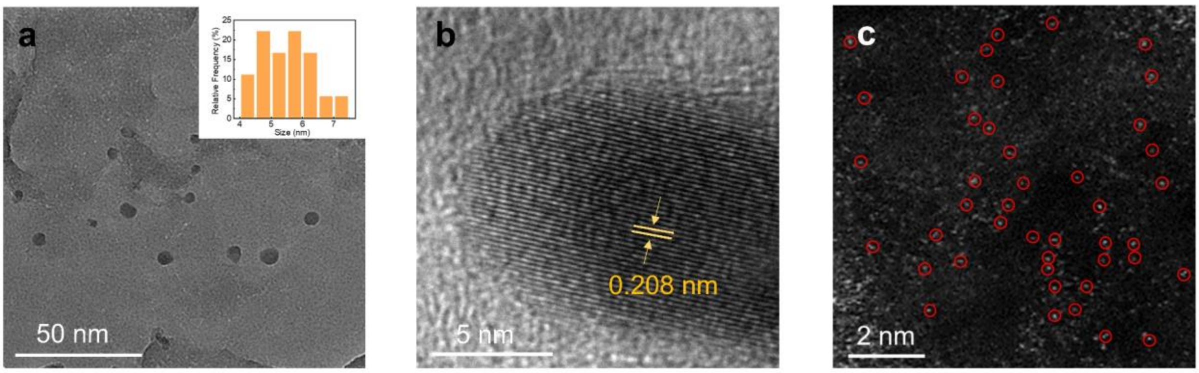


**Figure S2.** (a) TEM image (inset shows particle size distribution), (b) HRTEM image and (c) aberration-corrected HAADF-STEM image of R-$Cu_1/Cu_{NP}$.

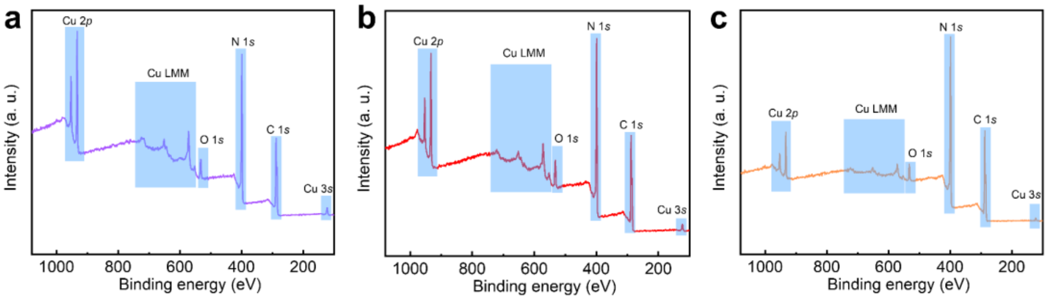


**Figure S3.** XPS survey spectra of (a) P-$Cu_1/Cu_{NP}$, (b) M-$Cu_1/Cu_{NP}$ and (c) R-$Cu_1/Cu_{NP}$.

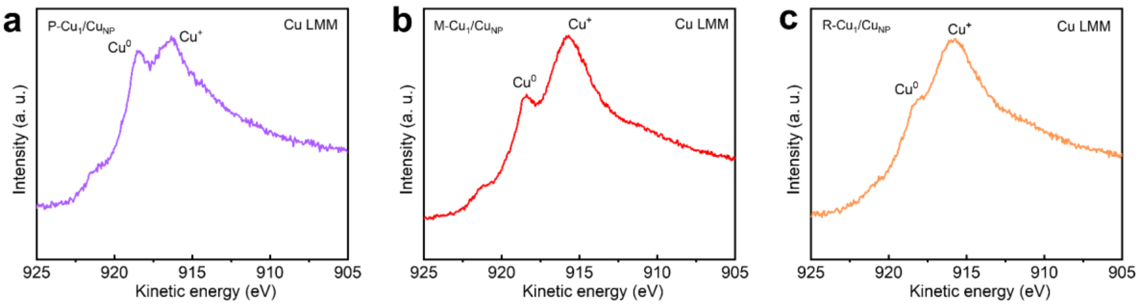


**Figure S4.** Cu Auger LMM spectra of (a) P-$Cu_1$/$Cu_{NP}$, (b) M-$Cu_1$/$Cu_{NP}$ and (c) R-$Cu_1$/$Cu_{NP}$.

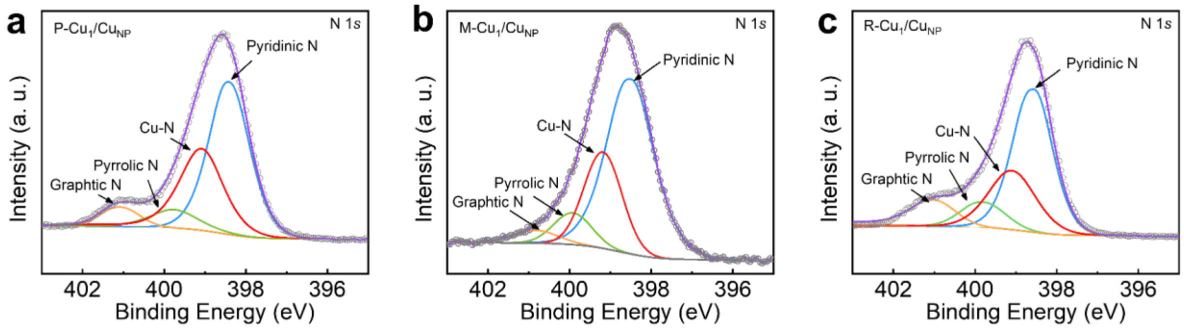


**Figure S5.** XPS spectra of N 1*s* orbits of (a) P-$Cu_1/Cu_{NP}$, (b) M-$Cu_1/Cu_{NP}$, and (c) R-$Cu_1/Cu_{NP}$.

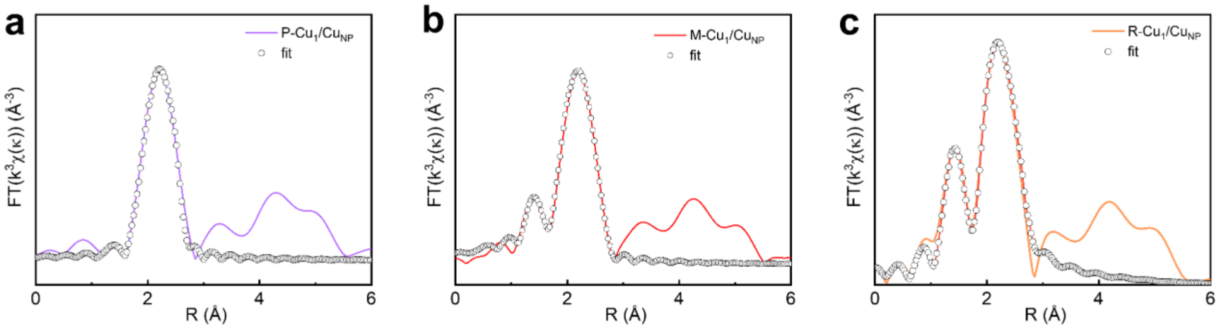


**Figure S6.** Least-squares EXAFS fittings of (a) P-$Cu_1$/$Cu_{NP}$, (b) M-$Cu_1$/$Cu_{NP}$, and (c) R-$Cu_1$/$Cu_{NP}$.

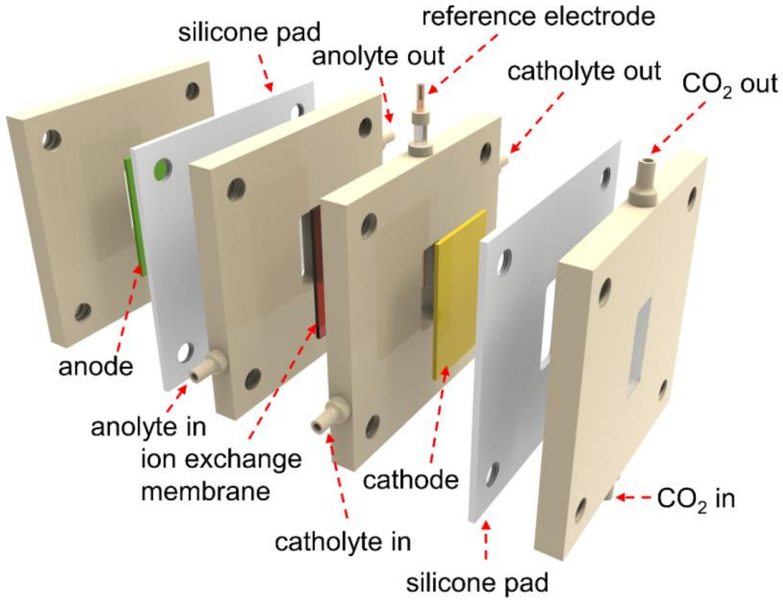


**Figure S7.** The scheme of flow cell used for $CO_2$RR tests.

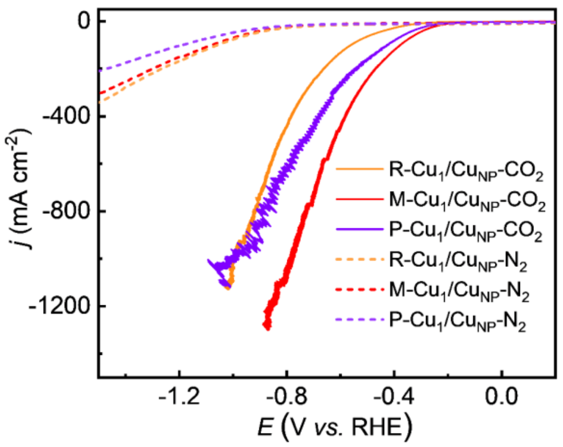


**Figure S8.** LSV curves of R-$Cu_1$/$Cu_{NP}$, M-$Cu_1$/$Cu_{NP}$ and P-$Cu_1$/$Cu_{NP}$ in $CO_2$ or $N_2$ atmosphere, the experiments were performed in 5 M KOH electrolyte with 20 mV $s^{-1}$ scan rate and 80% ohmic compensation.

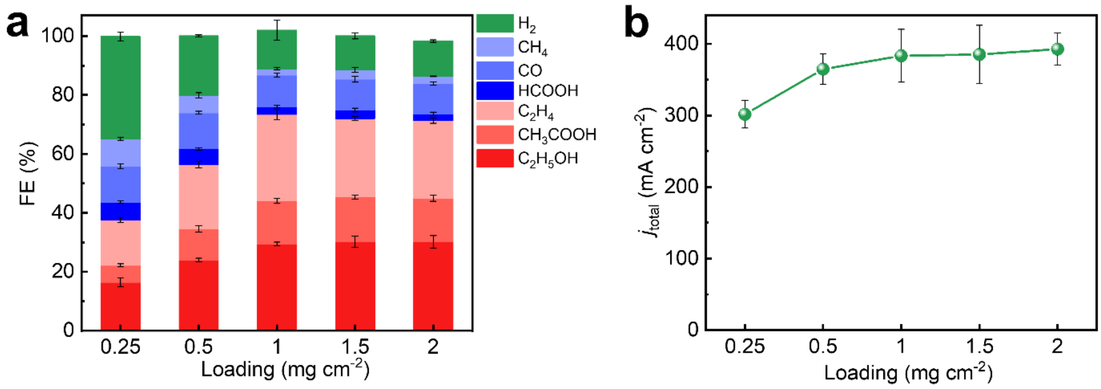


**Figure S9.** (a) Product FEs and (b) current density of M-$Cu_1$/$Cu_{NP}$ at -0.6V in 5 M KOH electrolyte with various catalyst loading. Both $C_2$ products selectivity and total current density increased as the increasing loading of M-$Cu_1$/$Cu_{NP}$ until 1 mg cm$^{-2}$, and no obvious difference was observed among 1, 1.5 and 2 mg cm$^{-2}$. Therefore, we chose 1 mg cm$^{-2}$ as the catalyst loading to conduct activity experiments.

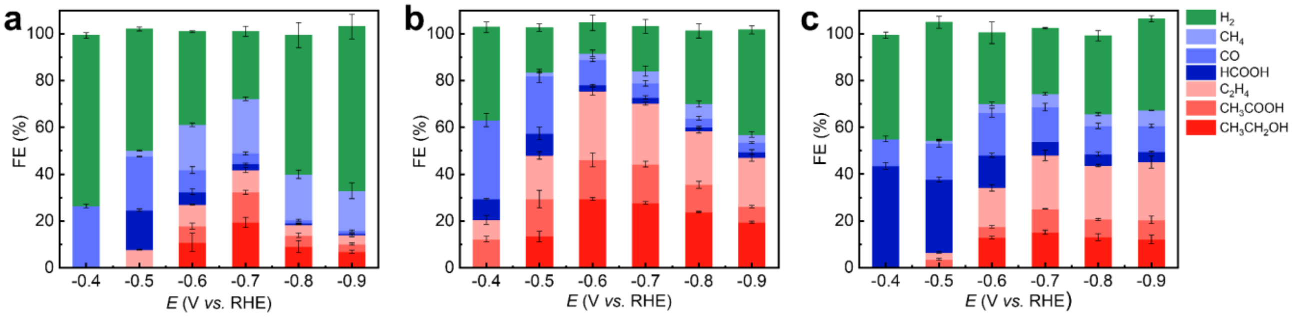


**Figure S10.** The $CO_2$RR prodcuct FEs of (a) R-$Cu_1$/$Cu_{NP}$, (b) M-$Cu_1$/$Cu_{NP}$ and (c) P-$Cu_1$/$Cu_{NP}$ under different applied potentials, the experiments were performed in 5 M KOH electrolyte with 80% ohmic compensation. Values are means and error bars indicate s.d. (*n* = 3 replicates).

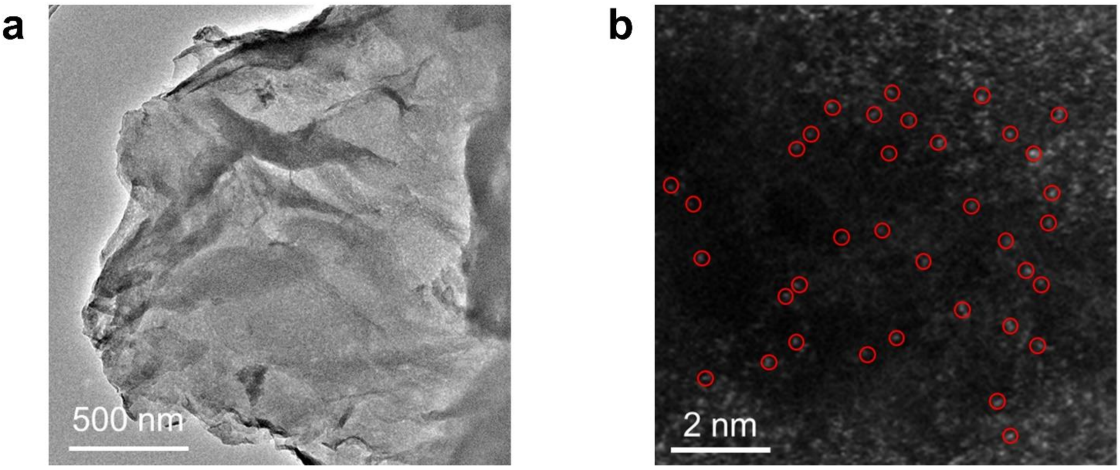


**Figure S11.** (a) TEM and (b) aberration-corrected HAADF-STEM images of Cu-N-C. The Cu-N-C was obtained by acid treatment of M-$Cu_1$/$Cu_{NP}$: the M-$Cu_1$/$Cu_{NP}$ was added into 50 mL 1 M sulfuric acid aqueous solution and heated at 80 °C for 48 h, then washed with deionized water several times and dried at 80°C overnight.

No obvious nanoparticles were observed in TEM image and a large amount of isolated bright dots recognized as Cu single atoms were observed in aberration-corrected HAADF-STEM image.

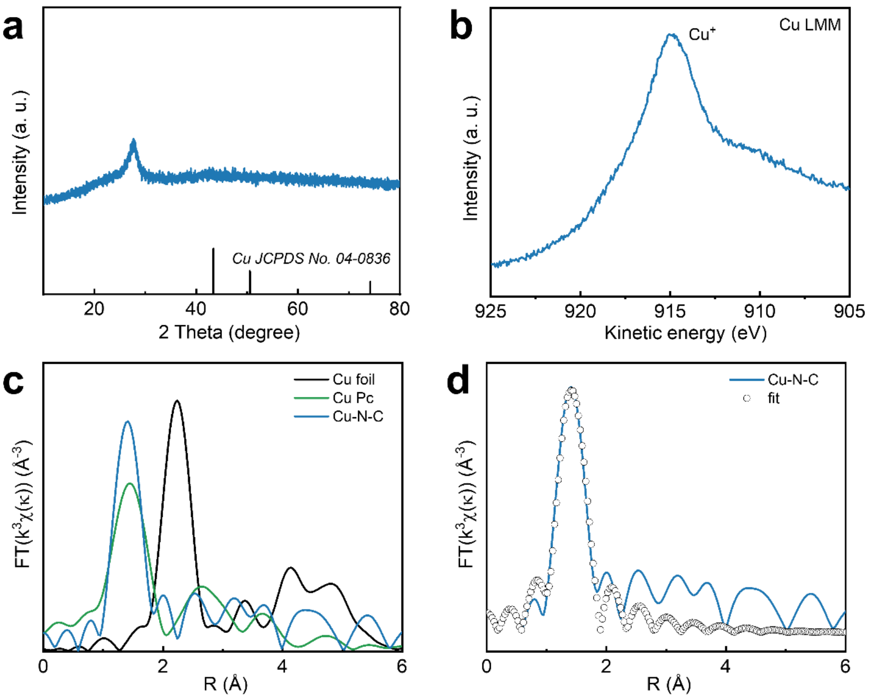


**Figure S12.** (a) XRD pattern, (b) Cu Auger LMM spectrum, (c) EXAFS spectra, and (d) least-squares EXAFS fittings of Cu-N-C. The Cu-N-C was obtained by acid treatment of M-$Cu_1/Cu_{NP}$: the M-$Cu_1/Cu_{NP}$ was added into 50 mL 1 M sulfuric acid aqueous solution and heated at 80 °C for 48 h, then washed with deionized water several times and dried at 80°C overnight.

No diffraction peaks related to crystalline Cu species can be identified in XRD pattern. XPS spectra showed that Cu element existed in Cu-N-C catalyst and $Cu^+$ was main specie. More importantly, the EXAFS profiles in the R-space of Cu-N-C suggested that only the peak attributed to Cu-N coordination located at around 1.4 Å, while the Cu-Cu coordination peak at around 2.2 Å disappeared, which confirmed that the Cu species only existed as single atom form. The results of least-squares EXAFS fitting confirmed that the Cu-N coordination numbers in Cu-N-C was 4.0 (Table S2), implying that the atomic Cu species mainly existed as Cu-$N_4$ structure.

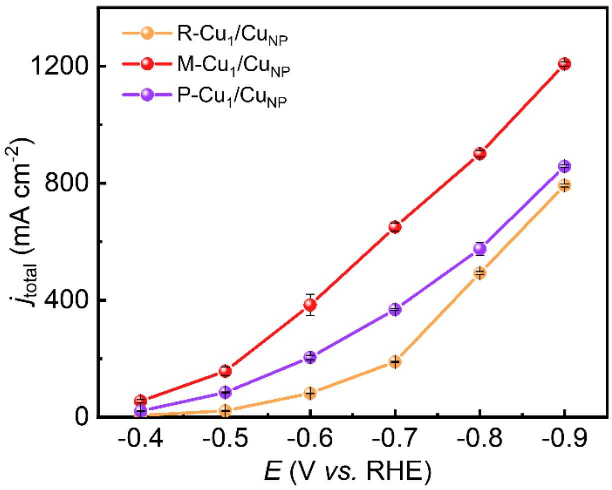


**Figure S13.** Total current density of R-$Cu_1$/$Cu_{NP}$, M-$Cu_1$/$Cu_{NP}$ and P-$Cu_1$/$Cu_{NP}$ at different applied potentials in 5 M KOH electrolyte. Values are means and error bars indicate s.d. ($n$ = 3 replicates).

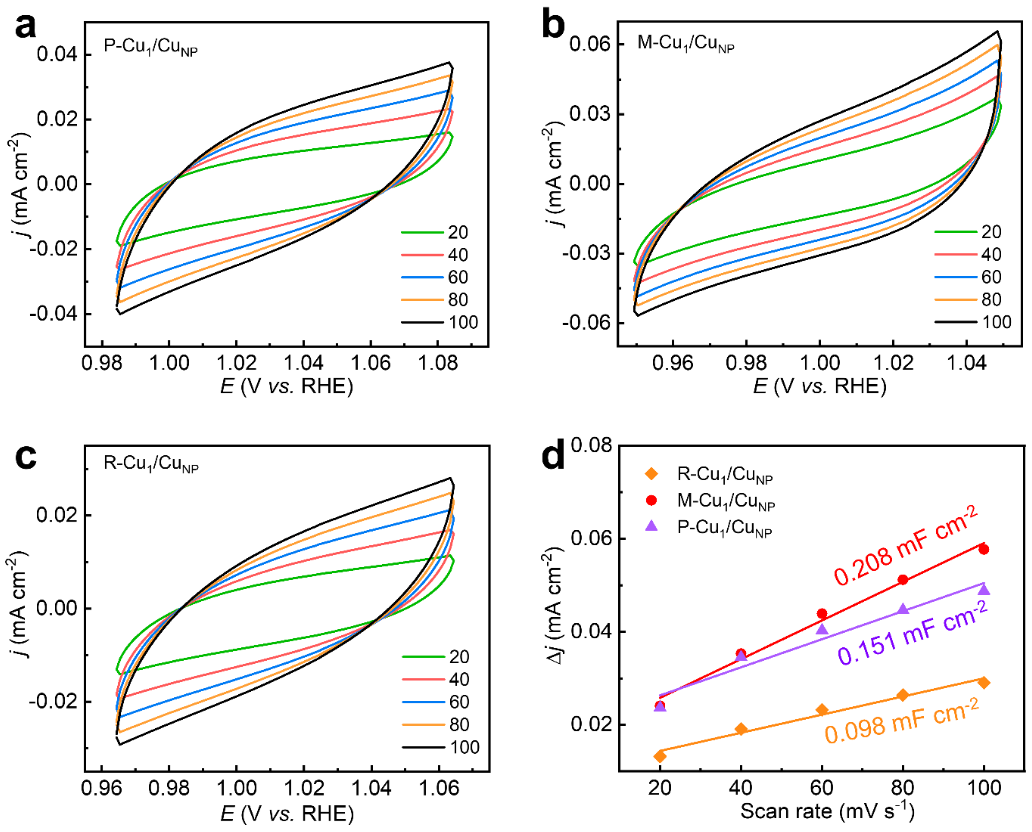


**Figure S14.** Cyclic voltammograms with different scan rates (20, 40, 60, 80, 100 mV $s^{-1}$) for (a) P-$Cu_1$/$Cu_{NP}$, (b) M-$Cu_1$/$Cu_{NP}$, and (c) R-$Cu_1$/$Cu_{NP}$ in 0.5 M $KHCO_3$ electrolyte. (d) Linear fitting of double-layer capacitive currents Δ*j* versus scan rate.

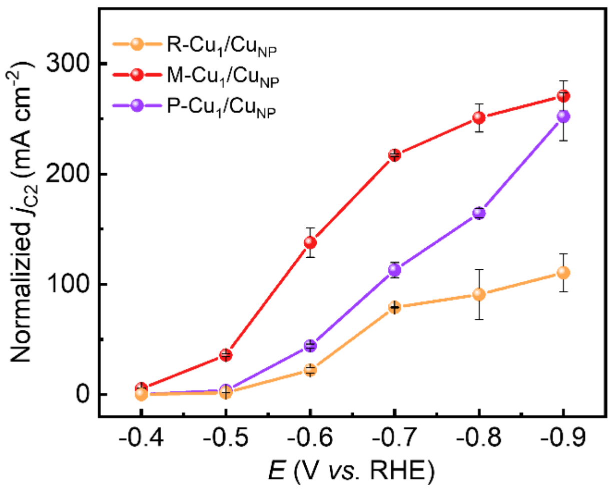


**Figure S15.** The $j_{C2}$ of R-$Cu_1$/$Cu_{NP}$, M-$Cu_1$/$Cu_{NP}$ and P-$Cu_1$/$Cu_{NP}$ after normalized by ECSA in 5 M KOH electrolyte.

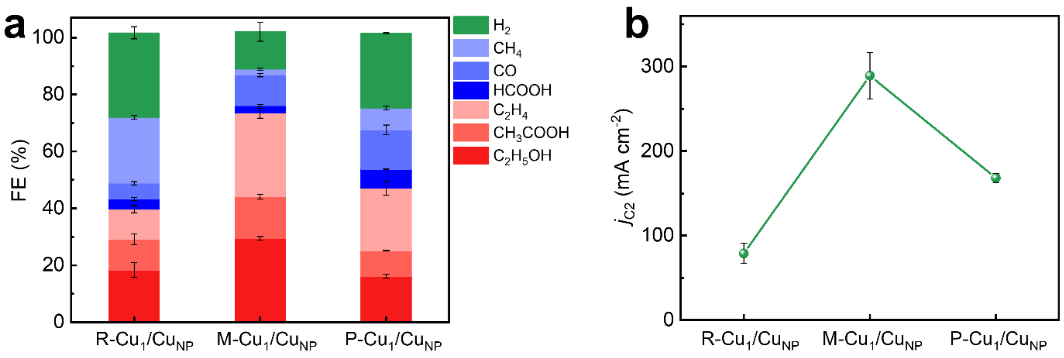


**Figure S16.** The (a) products FE and (b) $j_{C2}$ of R-$Cu_1$/$Cu_{NP}$, M-$Cu_1$/$Cu_{NP}$ and P-$Cu_1$/$Cu_{NP}$ under similar ECSA condition in 5 M KOH electrolyte.

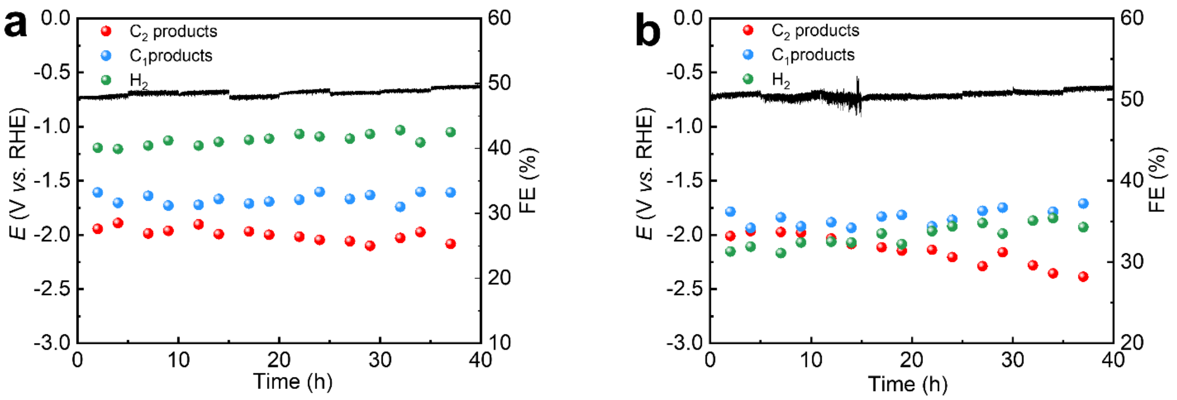


**Figure S17.** Long-term stability of (a) R-$Cu_1/Cu_{NP}$ at a constant current density of 200 mA $cm^{-2}$ and (b) P-$Cu_1/Cu_{NP}$ at a constant current density of 400 mA $cm^{-2}$ in 5 M KOH electrolyte (the electrode was washed, then dried and the electrolyte was refreshed at intervals 5 h to address the issues of flooding and carbonation).

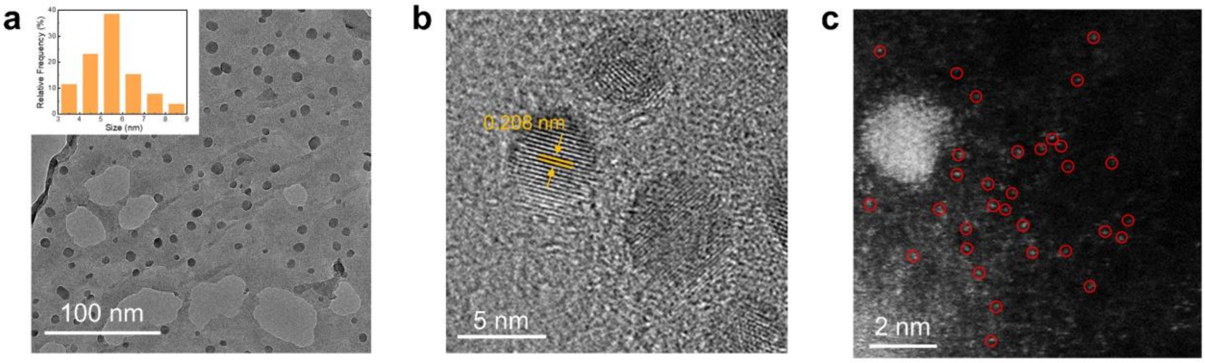


**Figure S18.** (a) TEM image (inset shows particle size distribution), (b) HRTEM image and (c) aberration-corrected HAADF-STEM image of M-$Cu_1/Cu_{NP}$ after 40 h electrolysis.

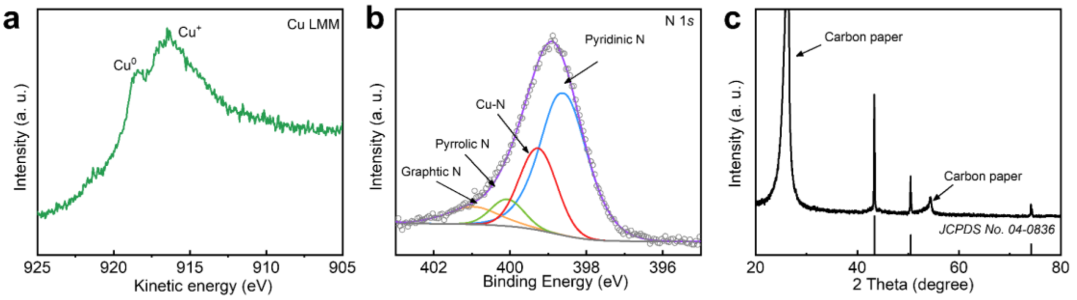


**Figure S19.** (a) Cu Auger LMM spectrum (b) XPS spectra of N *1s* orbits and (c) XRD pattern of M-$Cu_1$/$Cu_{NP}$ after 40 h electrolysis.

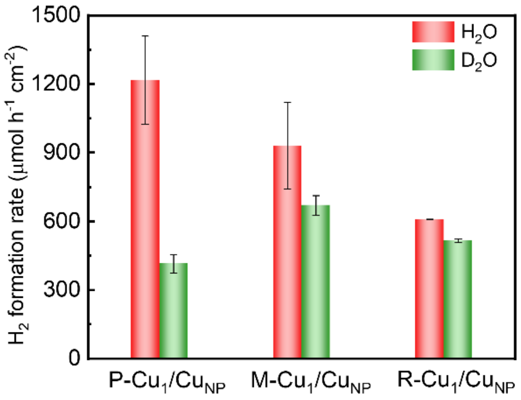


**Figure S20.** The kinetic isotope effect of $H_2O/D_2O$ on P-$Cu_1/Cu_{NP}$ M-$Cu_1/Cu_{NP}$ and R-$Cu_1/Cu_{NP}$ at -0.6 V in flow cell with 5 M KOH electrolyte. Values are means and error bars indicate s.d. ($n$ = 3 replicates).

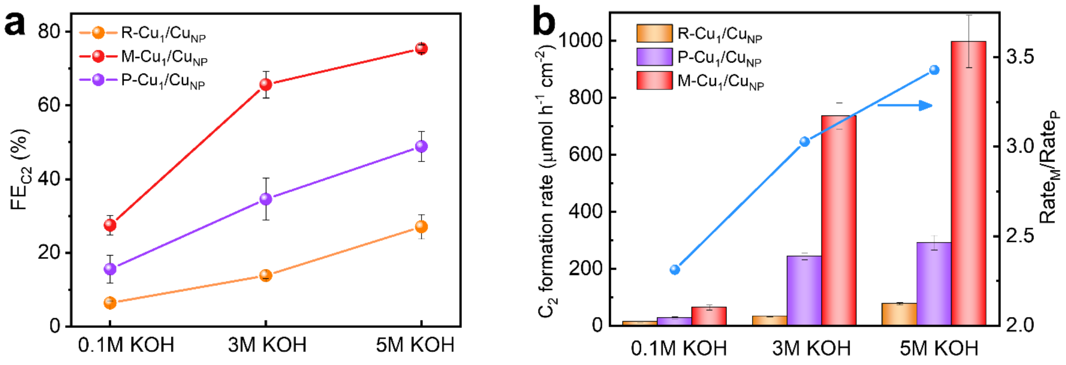


**Figure S21.** (a) $FE_{C2}$ of R-$Cu_1$/$Cu_{NP}$,M-$Cu_1$/$Cu_{NP}$ and P-$Cu_1$/$Cu_{NP}$ in different concentrations KOH electrolyte at -0.6 V. (b) $C_2$ formation rate of R-$Cu_1$/$Cu_{NP}$,M-$Cu_1$/$Cu_{NP}$ and P-$Cu_1$/$Cu_{NP}$ in different concentrations KOH electrolyte at -0.6 V and $Rate_M$/$Rate_P$ of M-$Cu_1$/$Cu_{NP}$ to P-$Cu_1$/$Cu_{NP}$. Values are means and error bars indicate s.d. ($n$ = 3 replicates).

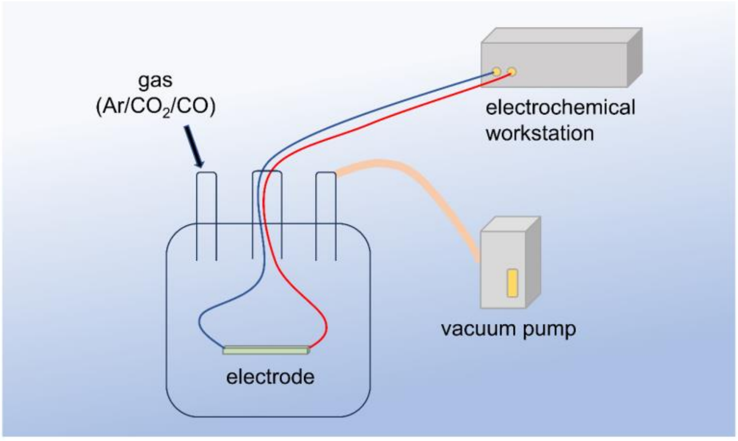


**Figure S22.** The device scheme of gas electro-response experiments.

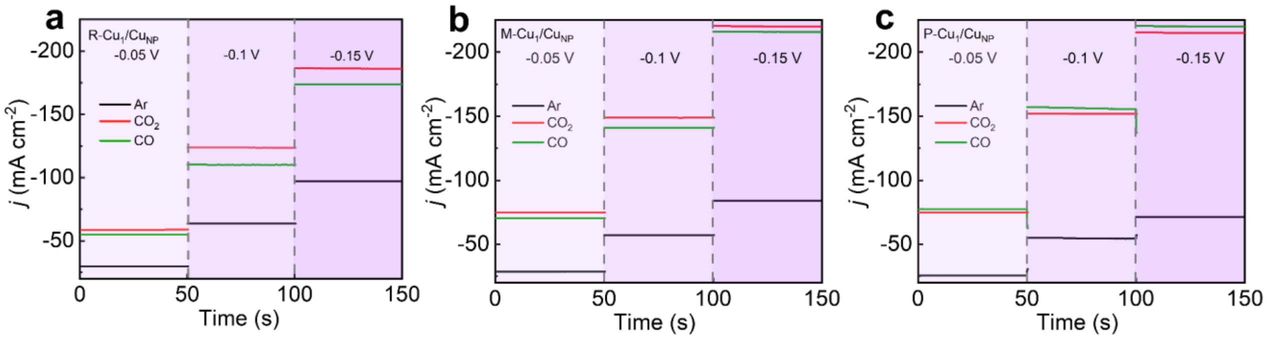


**Figure S23.** The $CO_2$ and CO gas adsorption electroresponse current density of (a) R-$Cu_1/Cu_{NP}$, (b) M-$Cu_1/Cu_{NP}$ and (c) P-$Cu_1/Cu_{NP}$.

The capacity adsorption of $CO_2$ and CO molecules on the catalysts surface was tested by a self-desined gas adsorption electroresponse device. The Cu foam sprayed with the catalyst was used as electrode. The as-prepared electrode was placed in a sealed container and connected with the electrochemical workstation. Various gas ($CO_2$、CO and Ar) were injected into the container and the curve of current was monitored under different applied potentials (-0.05, -0.1 and -0.15 V).

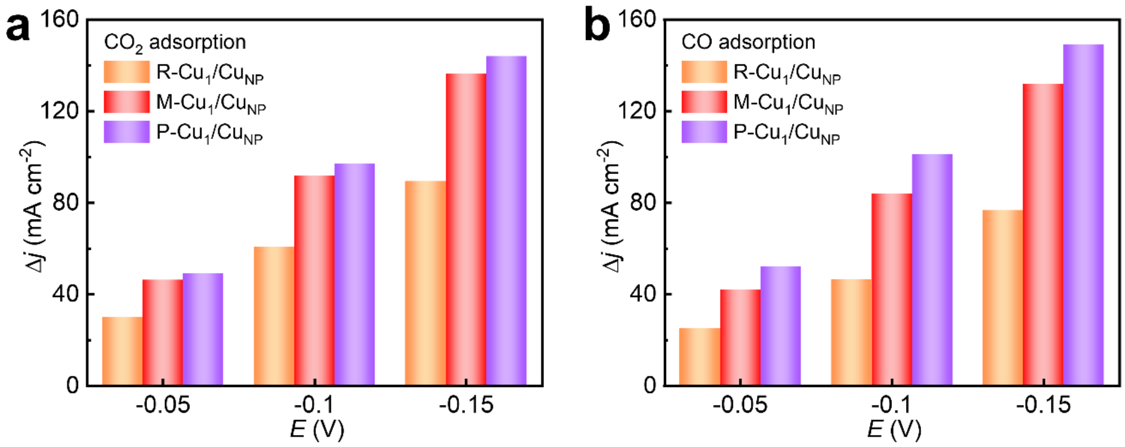


**Figure S24.** Results of (a) $CO_2$ and (b) CO adsorption responses under different applied voltages over R-$Cu_1$/$Cu_{NP}$, M-$Cu_1$/$Cu_{NP}$ and P-$Cu_1$/$Cu_{NP}$.

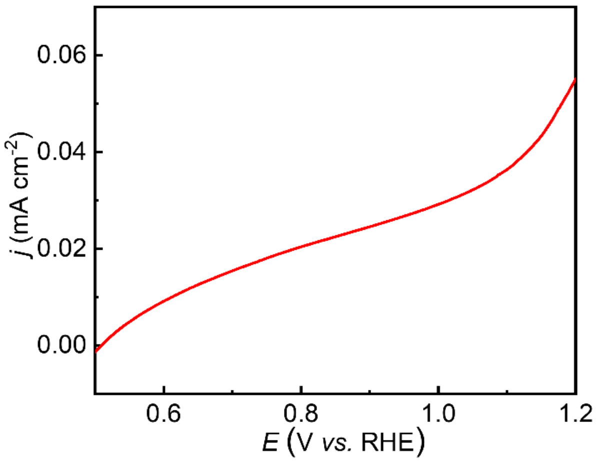


**Figure S25.** The LSV cruve of M-$Cu_1$/$Cu_{NP}$ in 0.1 M $Na_2SO_4$ aqueous solution without CO adsorption.

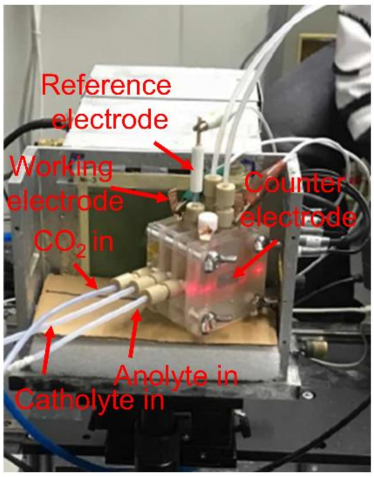


**Figure S26.** The optical photo of flow cell used for *in situ* XAS experiment.

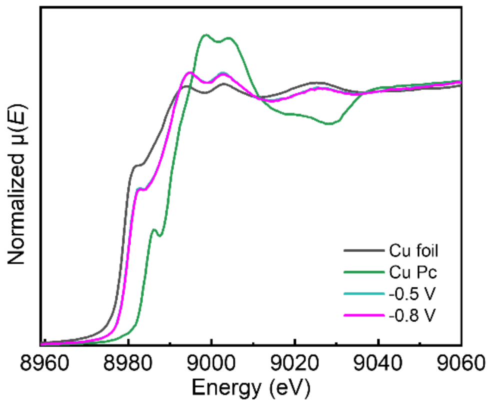


**Figure S27.** The *in situ* XANES spectra at Cu K-edge over M-$Cu_1$/$Cu_{NP}$ at -0.5 and -0.8 V in 5 M KOH electrolyte.

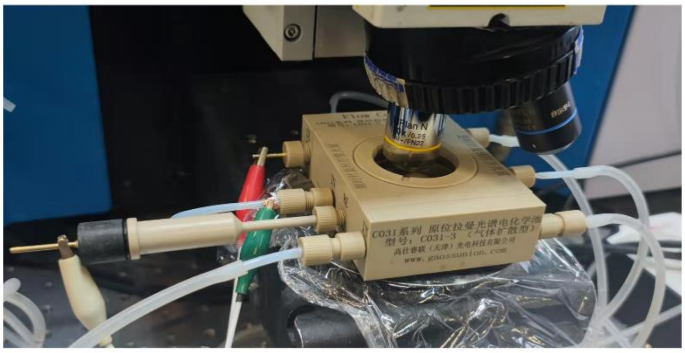


**Figure S28.** The photograph of cell used for *in situ* SERS spectroscopy.

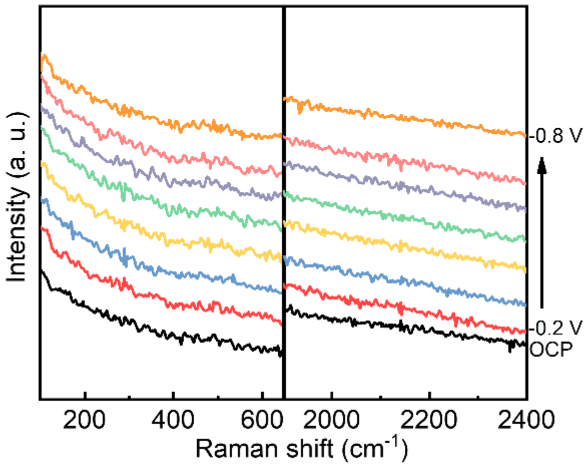


**Figure S29.** *In situ* surface-enhanced Raman spectra recorded at different applied potentials for Cu-N-C during $CO_2$RR.

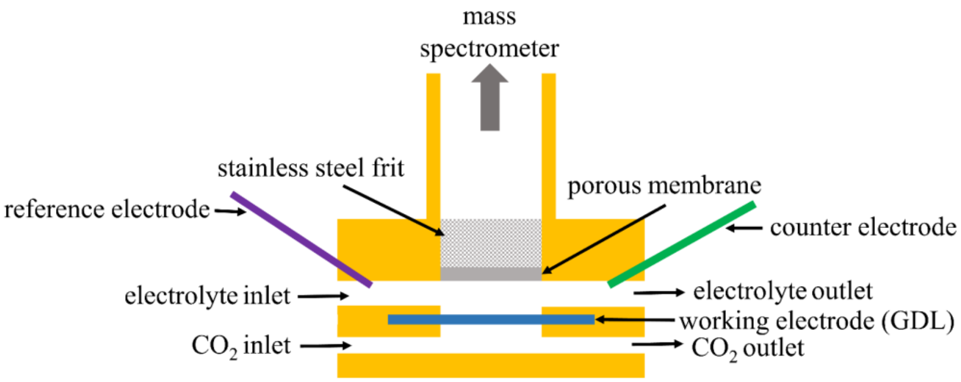


**Figure S30.** The scheme of cell used for online DEMS measurements.

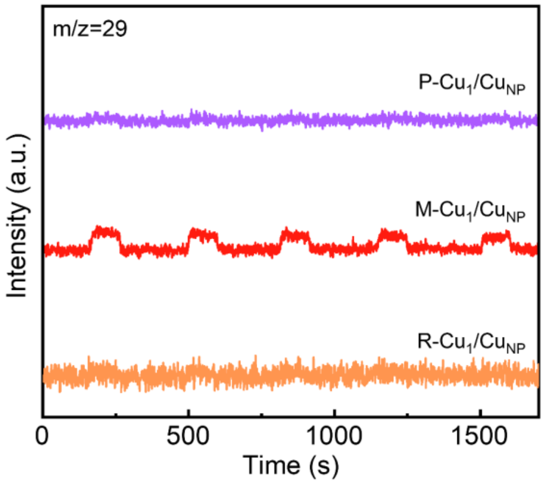


**Figure S31.** The m/z signal of 29 of P-$Cu_1$/$Cu_{NP}$, M-$Cu_1$/$Cu_{NP}$ and R-$Cu_1$/$Cu_{NP}$ during online DEMS measurements for $CO_2$RR at -0.6 V in five continuous cycles in 5 M KOH electrolyte.

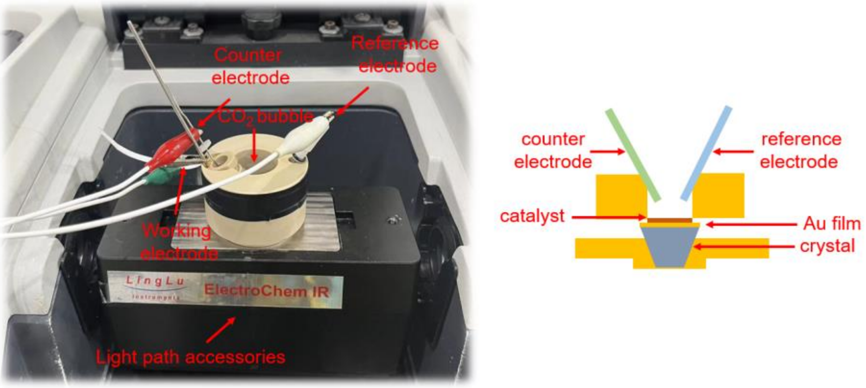


**Figure S32.** The photograph and scheme of cell used for *in situ* ATR-SEIRAS spectroscopy.

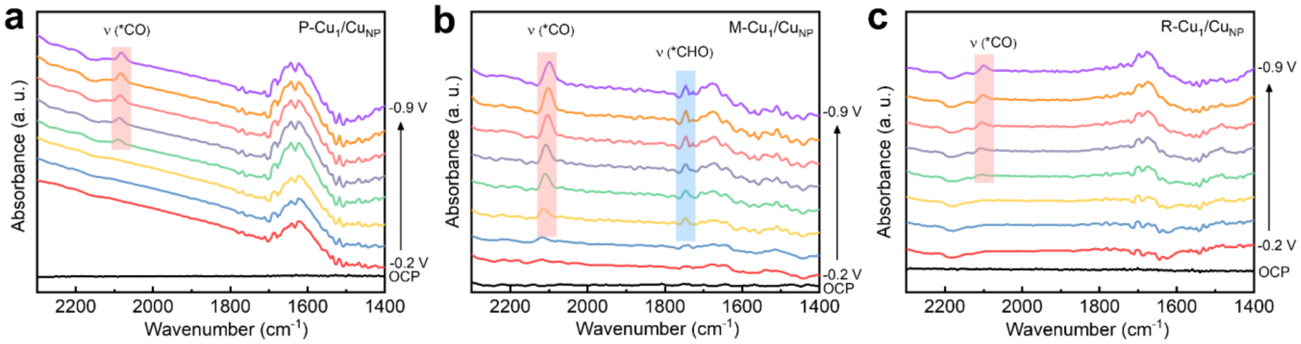


**Figure S33.** *In situ* ATR-SEIRAS spectra recorded at different applied potentials for (a) P-$Cu_1/Cu_{NP}$, (b) M-$Cu_1/Cu_{NP}$ and (c) R-$Cu_1/Cu_{NP}$ during $CO_2RR$ in $CO_2$-saturated 3 M KOH electrolyte.

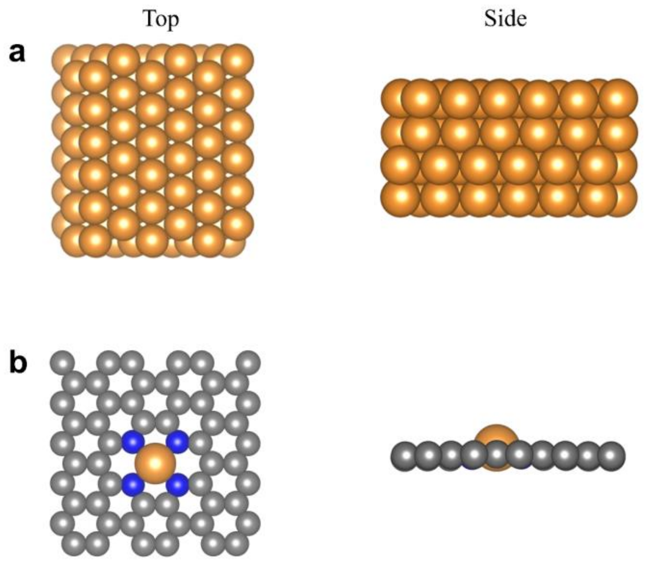


**Figure S34.** The top and side views of (a) Cu(111) and (b) Cu-$N_4$ models.

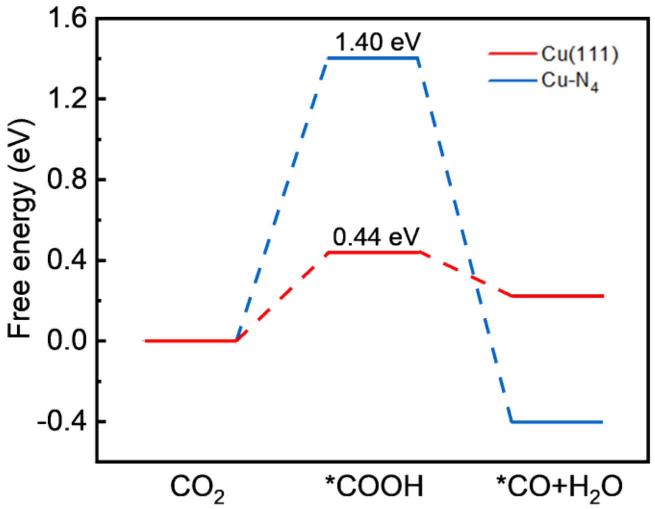


**Figure S35.** The free energy diagram for $CO_2$RR to describe the activation of $CO_2$ over Cu(111) and Cu-$N_4$.

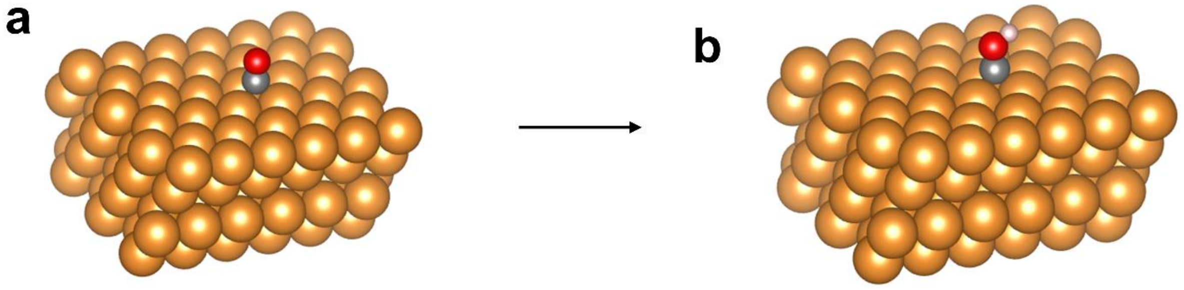


**Figure S36.** Side views of (a) *CO and (b) *COH on the Cu(111) facet.

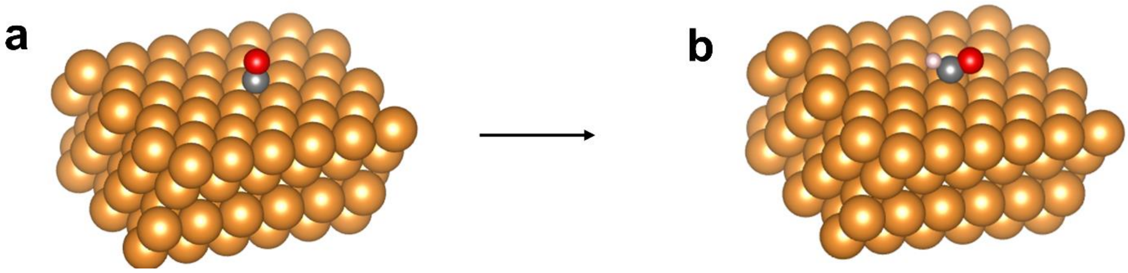


**Figure S37.** Side views of (a) *CO and (b) *CHO on the Cu(111) facet.

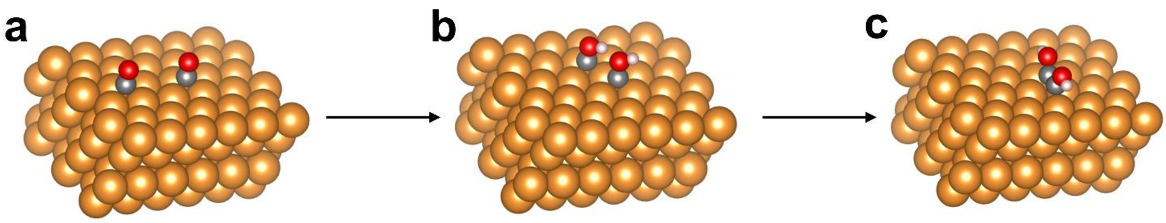


**Figure S38.** Side views of (a) *CO-*CO, (b) *COH-*COH and (c) *COH*COH on the Cu(111) facet.

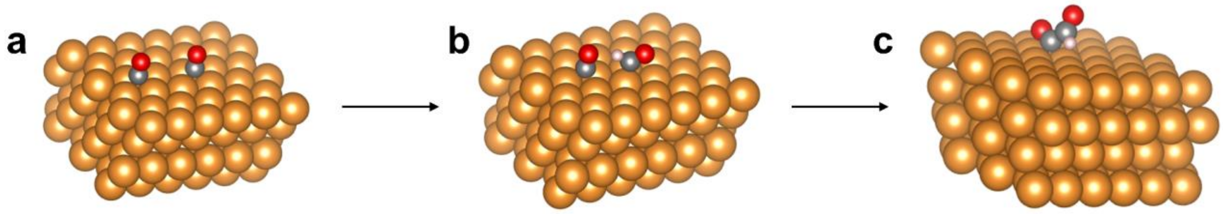


**Figure S39.** Side views of (a) *CO-*CO, (b) *CO-*CHO and (c) O*CCHO on the Cu(111) facet.

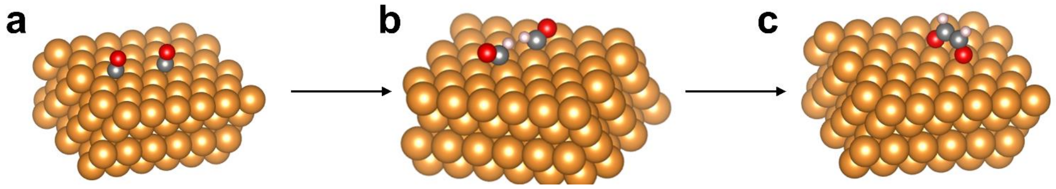


**Figure S40.** Side views of (a) *CO-*CO, (b) *CHO-*CHO and (c) *OHCCHO* on the Cu(111) facet.

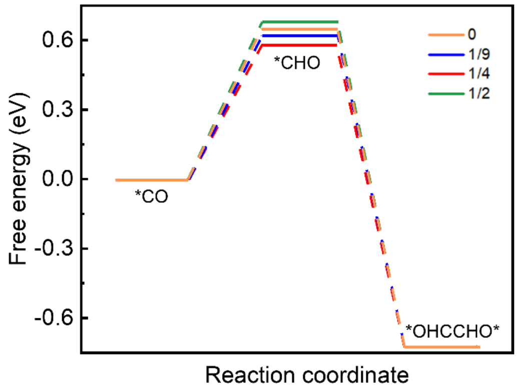


**Figure S41.** Free energy of *CO hydrogenation to *CHO on Cu(111) under different *H coverage.

**Table S1.** The contents of Cu, N and C of the catalysts measured by XPS

| Sample | Cu at% | N at% | C at% |
|---|---|---|---|
| P-$Cu_1$/$Cu_{NP}$ | 7.4 | 33.5 | 59.1 |
| M-$Cu_1$/$Cu_{NP}$ | 4.3 | 40.5 | 55.2 |
| R-$Cu_1$/$Cu_{NP}$ | 1.9 | 47.2 | 50.9 |

**Table S2.** Structural parameters extracted from the EXAFS fitting. ($S_0^2$=0.80)

| Sample | Scattering pair | $CN_{real}$ | R(Å) | $\sigma^2(10^{-3}Å^2)$ | $\Delta E_0$(eV) | P (%) |
|---|---|---|---|---|---|---|
| R-$Cu_1/Cu_{NP}$ | Cu-N | 3.9 | 1.96 | 5.8 | -3.5 | 28 ($Cu_1$) |
| | Cu-Cu | 8.6 | 2.56* | 4.9 | 2.1 | 72 ($Cu_{NP}$) |
| M-$Cu_1/Cu_{NP}$ | Cu-N | 3.8 | 1.96 | 5.1 | -3.4 | 20 ($Cu_1$) |
| | Cu-Cu | 8.6 | 2.56* | 5.4 | 2.1 | 80 ($Cu_{NP}$) |
| P-$Cu_1/Cu_{NP}$ | Cu-N | 0.4 | 1.96 | 6.0 | -3.5 | 5 ($Cu_1$) |
| | Cu-Cu | 8.4 | 2.56* | 4.8 | 2.1 | 95 ($Cu_{NP}$) |
| Cu-N-C | Cu-N | 4.0 | 1.96 | 5.9 | -3.5 | - |

$S_0^2$ is the amplitude reduction factor $S_0^2$=0.8; $CN_{real}$ is the coordination number; R is interatomic distance (the bond length between central atoms and surrounding coordination atoms); $\sigma^2$ is Debye-Waller factor (a measure of thermal and static disorder in absorber-scatterer distances); $\Delta E_0$ is edge-energy shift (the difference between the zero kinetic energy value of the sample and that of the theoretical model). R factor is used to value the goodness of the fitting. * represents a fixed value for the parameter. P represents the percentage of $Cu-N_4$ ($Cu_1$) and Cu NPs Cu in total Cu species of catalyst.

**Table S3.** Product FEs, total current density and error bar of R-$Cu_1/Cu_{NP}$ at various potentials in 5 M KOH electrolyte.

| Potential (V) | $C_2H_5OH$ FE (%) | | | Error bar |
|---|---|---|---|---|
| -0.4 | 0.0 | 0.0 | 0.0 | 0.00 |
| -0.5 | 0.0 | 0.0 | 0.0 | 0.00 |
| -0.6 | 14.8 | 11.3 | 7.0 | 3.90 |
| -0.7 | 17.2 | 21.6 | 19.2 | 2.20 |
| -0.8 | 9.0 | 11.7 | 6.4 | 2.65 |
| -0.9 | 6.3 | 7.1 | 7.5 | 0.61 |

| Potential (V) | $CH_3COOH$ FE (%) | | | Error bar |
|---|---|---|---|---|
| -0.4 | 0.0 | 0.0 | 0.0 | 0.00 |
| -0.5 | 0.0 | 0.0 | 0.0 | 0.00 |
| -0.6 | 5.5 | 8.2 | 6.8 | 1.35 |
| -0.7 | 12.7 | 12.0 | 13.7 | 0.85 |
| -0.8 | 3.8 | 5.7 | 4.7 | 0.95 |
| -0.9 | 2.8 | 3.3 | 3.7 | 0.45 |

| Potential (V) | $C_2H_4$ FE (%) | | | Error bar |
|---|---|---|---|---|
| -0.4 | 0.0 | 0.0 | 0.0 | 0.00 |
| -0.5 | 7.6 | 7.7 | 7.9 | 0.15 |
| -0.6 | 9.1 | 9.5 | 9.2 | 0.21 |
| -0.7 | 9.6 | 8.5 | 10.6 | 1.05 |
| -0.8 | 4.8 | 4.5 | 4.5 | 0.17 |
| -0.9 | 3.2 | 4.4 | 4.0 | 0.61 |

| Potential (V) | HCOOH FE (%) | | | Error bar |
|---|---|---|---|---|
| -0.4 | 0.0 | 0.0 | 0.0 | 0.00 |
| -0.5 | 17.4 | 17.1 | 16.4 | 0.51 |
| -0.6 | 4.1 | 6.7 | 5.5 | 1.30 |
| -0.7 | 2.7 | 2.1 | 3.0 | 0.46 |
| -0.8 | 0.5 | 0.7 | 0.6 | 0.10 |
| -0.9 | 0.5 | 0.6 | 0.7 | 0.10 |

| Potential (V) | CO FE (%) | | | Error bar |
|---|---|---|---|---|
| -0.4 | 22.3 | 23.1 | 21.4 | 0.85 |
| -0.5 | 23.1 | 22.8 | 23 | 0.15 |
| -0.6 | 7.9 | 10.8 | 9.2 | 1.45 |
| -0.7 | 4 | 4.5 | 5.1 | 0.55 |
| -0.8 | 0.8 | 1.8 | 1.2 | 0.50 |
| -0.9 | 1.2 | 1.6 | 0.9 | 0.35 |

| Potential (V) | $CH_4$ FE (%) | | | Error bar |
|---|---|---|---|---|
| -0.4 | 0.0 | 0.0 | 0.0 | 0.00 |
| -0.5 | 2.8 | 2.2 | 2.4 | 0.31 |
| -0.6 | 19.4 | 18.6 | 20.0 | 0.70 |
| -0.7 | 22.6 | 24.0 | 22.9 | 0.74 |
| -0.8 | 17.8 | 21.3 | 20.0 | 1.77 |
| -0.9 | 13.7 | 20.5 | 17.5 | 3.41 |

| Potential (V) | $H_2$ FE (%) | | | Error bar |
|---|---|---|---|---|
| -0.4 | 71.8 | 73.7 | 74.0 | 1.19 |
| -0.5 | 51.1 | 52.7 | 52.1 | 0.81 |
| -0.6 | 40.2 | 39.5 | 40.1 | 0.38 |
| -0.7 | 30.9 | 28.5 | 26.6 | 2.15 |
| -0.8 | 64.8 | 54.2 | 59.8 | 5.30 |
| -0.9 | 76.4 | 64.0 | 70.5 | 6.20 |

| Potential (V) | Total current density (mA $cm^{-2}$) | | | Error bar |
|---|---|---|---|---|
| -0.4 | 5.7 | 6.2 | 6.0 | 0.25 |
| -0.5 | 21.3 | 22.9 | 19.8 | 1.55 |
| -0.6 | 81.1 | 82.3 | 81.5 | 0.60 |
| -0.7 | 191.0 | 187.0 | 189.0 | 2.00 |
| -0.8 | 493.2 | 487.0 | 497.2 | 5.14 |
| -0.9 | 798.0 | 787.0 | 792.0 | 5.51 |

**Table S4.** Product FEs, total current density and error bar of M-$Cu_1/Cu_{NP}$ at various potentials in 5 M KOH electrolyte.

| Potential (V) | $C_2H_5OH$ FE (%) | | | Error bar |
|---|---|---|---|---|
| -0.4 | 0 | 0 | 0 | 0 |
| -0.5 | 10.8 | 15.3 | 14 | 2.30 |
| -0.6 | 30.1 | 29.2 | 29 | 0.59 |
| -0.7 | 27 | 28 | 28 | 0.58 |
| -0.8 | 24.1 | 23.9 | 23.6 | 0.25 |
| -0.9 | 19.6 | 19.9 | 19 | 0.46 |

| Potential (V) | $CH_3COOH$ FE (%) | | | Error bar |
|---|---|---|---|---|
| -0.4 | 12.7 | 13.1 | 10.9 | 1.17 |
| -0.5 | 18.3 | 11.8 | 18 | 3.67 |
| -0.6 | 15.5 | 14.4 | 20 | 2.97 |
| -0.7 | 17.7 | 16.7 | 15.3 | 1.21 |
| -0.8 | 11.8 | 13.1 | 10 | 1.56 |
| -0.9 | 7.2 | 6.3 | 6.5 | 0.47 |

| Potential (V) | $C_2H_4$ FE (%) | | | Error bar |
|---|---|---|---|---|
| -0.4 | 6.9 | 7.3 | 10.5 | 1.97 |
| -0.5 | 17.6 | 17.9 | 20.5 | 1.59 |
| -0.6 | 29.8 | 30.8 | 27.5 | 1.69 |
| -0.7 | 26.7 | 25.7 | 25.4 | 0.68 |
| -0.8 | 22.1 | 23.9 | 22.9 | 0.90 |
| -0.9 | 22.6 | 20.4 | 19.7 | 1.51 |

| Potential (V) | HCOOH FE (%) | | | Error bar |
|---|---|---|---|---|
| -0.4 | 8.6 | 8.9 | 9.2 | 0.30 |
| -0.5 | 12.5 | 7.5 | 7.9 | 2.78 |
| -0.6 | 2.4 | 3.1 | 2.5 | 0.38 |
| -0.7 | 3.5 | 2.2 | 2.3 | 0.72 |
| -0.8 | 1.5 | 2.1 | 1.5 | 0.35 |
| -0.9 | 1.2 | 2.7 | 3.2 | 1.04 |

| Potential (V) | CO FE (%) | | | Error bar |
|---|---|---|---|---|
| -0.4 | 35.3 | 35.6 | 30.4 | 2.92 |
| -0.5 | 23.7 | 27.8 | 22.4 | 2.82 |
| -0.6 | 10.1 | 11.3 | 10.7 | 0.60 |
| -0.7 | 7.4 | 5.4 | 5.3 | 1.18 |
| -0.8 | 3.2 | 3.4 | 4.7 | 0.81 |
| -0.9 | 4.1 | 3.6 | 4.5 | 0.45 |

| Potential (V) | $CH_4$ FE (%) | | | Error bar |
|---|---|---|---|---|
| -0.4 | 0 | 0 | 0 | 0.00 |
| -0.5 | 1.6 | 1.3 | 1.9 | 0.30 |
| -0.6 | 2.2 | 1.7 | 4.5 | 1.49 |
| -0.7 | 2.9 | 7.3 | 5.3 | 2.20 |
| -0.8 | 4.9 | 5.8 | 7.6 | 1.37 |
| -0.9 | 4.5 | 3.7 | 2.2 | 1.17 |

| Potential (V) | $H_2$ FE (%) | | | Error bar |
|---|---|---|---|---|
| -0.4 | 37.4 | 39.8 | 41.8 | 2.20 |
| -0.5 | 18.3 | 20.9 | 17.8 | 1.66 |
| -0.6 | 11.1 | 11.3 | 17.0 | 3.35 |
| -0.7 | 17.3 | 17.7 | 22.6 | 2.95 |
| -0.8 | 33.8 | 27.9 | 31.7 | 2.99 |
| -0.9 | 42.8 | 46 | 45.3 | 1.68 |

| Potential (V) | Total current density (mA $cm^{-2}$) | | | Error bar |
|---|---|---|---|---|
| -0.4 | 58.4 | 49.5 | 56.5 | 4.69 |
| -0.5 | 166.8 | 163.6 | 140 | 14.64 |
| -0.6 | 426 | 365.5 | 358.7 | 37.05 |
| -0.7 | 635.2 | 650.5 | 663.5 | 14.17 |
| -0.8 | 895.6 | 913.5 | 892.7 | 11.27 |
| -0.9 | 1211.83 | 1212.3 | 1198.8 | 7.66 |

**Table S5.** Product FEs, total current density and error bar of P-$Cu_1/Cu_{NP}$ at various potentials in 5 M KOH electrolyte.

| Potential (V) | $C_2H_5OH$ FE (%) | | | Error bar |
|---|---|---|---|---|
| -0.4 | 0.0 | 0.0 | 0.0 | 0.00 |
| -0.5 | 0.0 | 0.0 | 0.0 | 0.00 |
| -0.6 | 13.4 | 12.3 | 12.6 | 0.57 |
| -0.7 | 14.2 | 16.0 | 15.2 | 0.90 |
| -0.8 | 14.5 | 13.1 | 11.6 | 1.45 |
| -0.9 | 12.2 | 10.2 | 13.9 | 1.85 |

| Potential (V) | $CH_3COOH$ FE (%) | | | Error bar |
|---|---|---|---|---|
| -0.4 | 0.0 | 0.0 | 0.0 | 0.00 |
| -0.5 | 3.5 | 3.9 | 3.2 | 0.35 |
| -0.6 | 4.0 | 4.5 | 5.1 | 0.55 |
| -0.7 | 10.0 | 10.1 | 10.3 | 0.15 |
| -0.8 | 8.0 | 7.2 | 7.5 | 0.40 |
| -0.9 | 8.3 | 6.7 | 9.9 | 1.60 |

| Potential (V) | $C_2H_4$ FE (%) | | | Error bar |
|---|---|---|---|---|
| -0.4 | 0.0 | 0.0 | 0.0 | 0.00 |
| -0.5 | 3.1 | 2.9 | 3.5 | 0.31 |
| -0.6 | 16.2 | 17.2 | 14.5 | 1.37 |
| -0.7 | 21.1 | 24.7 | 20.3 | 2.34 |
| -0.8 | 23.0 | 23.2 | 24.0 | 0.53 |
| -0.9 | 27.8 | 23.9 | 22.9 | 2.59 |

| Potential (V) | HCOOH FE (%) | | | Error bar |
|---|---|---|---|---|
| -0.4 | 42.0 | 45.0 | 44.0 | 1.53 |
| -0.5 | 30.1 | 32.2 | 31.0 | 1.05 |
| -0.6 | 13.4 | 14.6 | 15.0 | 0.83 |
| -0.7 | 5.7 | 5.7 | 5.7 | 0.00 |
| -0.8 | 5.7 | 3.9 | 4.5 | 0.92 |
| -0.9 | 4.2 | 4.5 | 4.0 | 0.25 |

| Potential (V) | CO FE (%) | | | Error bar |
|---|---|---|---|---|
| -0.4 | 10.6 | 11.4 | 13.0 | 1.22 |
| -0.5 | 16.9 | 14.1 | 17.0 | 1.65 |
| -0.6 | 16.9 | 17.0 | 20.4 | 1.99 |
| -0.7 | 16.5 | 13.2 | 14.7 | 1.65 |
| -0.8 | 12.0 | 11.1 | 13.5 | 1.21 |
| -0.9 | 11.6 | 10.8 | 12.2 | 0.70 |

| Potential (V) | $CH_4$ FE (%) | | | Error bar |
|---|---|---|---|---|
| -0.4 | 0.0 | 0.0 | 0.0 | 0.00 |
| -0.5 | 1.0 | 1.2 | 1.4 | 0.20 |
| -0.6 | 4.5 | 3.1 | 3.4 | 0.74 |
| -0.7 | 5.9 | 5.1 | 6.2 | 0.57 |
| -0.8 | 4.5 | 5.5 | 5.5 | 0.58 |
| -0.9 | 6.7 | 6.6 | 6.5 | 0.10 |

| Potential (V) | $H_2$ FE (%) | | | Error bar |
|---|---|---|---|---|
| -0.4 | 45.6 | 43.1 | 44.0 | 1.27 |
| -0.5 | 48.1 | 53.1 | 51.1 | 2.52 |
| -0.6 | 30.5 | 28.2 | 37.1 | 4.62 |
| -0.7 | 28.4 | 28.0 | 28.5 | 0.26 |
| -0.8 | 31.5 | 33.7 | 35.8 | 2.15 |
| -0.9 | 40.4 | 38.1 | 40.5 | 1.36 |

| Potential (V) | Total current density (mA $cm^{-2}$) | | | Error bar |
|---|---|---|---|---|
| -0.4 | 21.2 | 19.8 | 20.4 | 0.70 |
| -0.5 | 83.4 | 85.9 | 84.3 | 1.27 |
| -0.6 | 211.2 | 196.0 | 204.5 | 7.62 |
| -0.7 | 367.0 | 365.7 | 370.0 | 2.21 |
| -0.8 | 554.0 | 598.3 | 573.2 | 22.22 |
| -0.9 | 860.1 | 860.0 | 852.0 | 4.65 |

**Table S6.** Product FEs, total current density and error bar of Cu-N-C at various potentials in 5 M KOH electrolyte.

| Potential (V) | CO FE (%) | | | Error bar |
|---|---|---|---|---|
| -0.4 | 0 | 0 | 0 | 0 |
| -0.5 | 3.1 | 2.7 | 2.5 | 0.30 |
| -0.6 | 2.4 | 3.4 | 2.4 | 0.57 |
| -0.7 | 2 | 3.5 | 2 | 0.86 |
| -0.8 | 1.3 | 3.2 | 1.4 | 1.06 |
| -0.9 | 0 | 1.8 | 0 | 1.03 |

| Potential (V) | $CH_4$ FE (%) | | | Error bar |
|---|---|---|---|---|
| -0.4 | 0 | 0 | 0 | 0.00 |
| -0.5 | 2.7 | 4.3 | 4.4 | 0.95 |
| -0.6 | 2.1 | 7.8 | 4.4 | 2.87 |
| -0.7 | 3.2 | 8.8 | 3.8 | 3.07 |
| -0.8 | 1.4 | 8.6 | 2.7 | 3.84 |
| -0.9 | 0 | 1.4 | 1.2 | 0.76 |

| Potential (V) | $H_2$ FE (%) | | | Error bar |
|---|---|---|---|---|
| -0.4 | 98.1 | 98.2 | 98.5 | 0.21 |
| -0.5 | 93.8 | 92.0 | 94.2 | 1.17 |
| -0.6 | 95.1 | 87.8 | 93.3 | 3.80 |
| -0.7 | 95.4 | 87.9 | 92.1 | 3.76 |
| -0.8 | 98.4 | 87.4 | 95.7 | 5.73 |
| -0.9 | 99.8 | 97.6 | 98.3 | 1.12 |

| Potential (V) | Total current density (mA $cm^{-2}$) | | | Error bar |
|---|---|---|---|---|
| -0.4 | 33.3 | 32.6 | 27.9 | 2.95 |
| -0.5 | 81.1 | 64.7 | 73.7 | 8.19 |
| -0.6 | 114.3 | 99.0 | 101.8 | 8.14 |
| -0.7 | 153.2 | 136.4 | 149.4 | 8.82 |
| -0.8 | 196.2 | 168.2 | 187.0 | 14.26 |
| -0.9 | 249.2 | 228.3 | 241.0 | 10.52 |

**Table S7.** Performance comparison of various catalysts for $CO_2$ electroreduction to $C_{2+}$ products.

| Samples | E (V *vs.* RHE) | $FE_{C2+}$ (%) | $j_{C2+}$ (mA $cm^{-2}$) | References |
|---|---|---|---|---|
| M-$Cu_1$/$Cu_{NP}$ | -0.60 | 75.4 | 289.2 | This work |
| Nanoporous Cu | -0.67 | 62.0 | 404.8 | 1 |
| Reconstructed Cu | -1.80 | 77.0 | 346.5 | 2 |
| NGQ/Cu-nr | -0.90 | ~74.0 | ~208.0 | 3 |
| F-Cu | -0.89 | 80.0 | 1280.0 | 4 |
| Multihollow $Cu_2O$ | -0.61 | 75.0 | 267.0 | 5 |
| CuAg wire | -0.70 | 85.0 | 255.0 | 6 |
| FeTPP[Cl]/Cu | -0.82 | 85.0 | 257.0 | 7 |
| OD-Cu-III | - | 74.9 | 224.7 | 8 |
| Cu dendrites | -0.68 | 64.0 | 255.0 | 9 |
| Cu | - | 80.4 | 120.6 | 10 |
| Cu(0)@PIL@Cuba(I) | -0.85 | 76.1 | 304.2 | 11 |
| 3-shell HoMSs | -0.88 | 77.0 | 513.7 | 12 |
| Cu-PTFE | -1.50 | 86.0 | ~250.0 | 13 |
| CuS/Cu-V | -0.92 | 52.8 | 147.8 | 14 |
| $Ce(OH)_x$-doped-Cu | -0.70 | 80.3 | 211.2 | 15 |
| Cu-KI | -1.09 | ~72.6 | 29.0 | 16 |
| Graphite/Cu/PTFE | -0.54 | 83.0 | 275.0 | 17 |
| Polyamine-incorporated Cu | -0.47 | 87.0 | ~35.0 | 18 |

## Supplementary References


1. Lv J. J. et al. A highly porous copper electrocatalyst for carbon dioxide reduction. *Adv. Mater.* **30**, 1803111 (2018).
2. Kibria M. G. et al. A surface reconstruction route to high productivity and selectivity in $CO_2$ electroreduction toward $C_{2+}$ hydrocarbons. *Adv. Mater.* **30**, 1804867 (2018).
3. Chen C. et al. Highly efficient electroreduction of $CO_2$ to $C_{2+}$ alcohols on heterogeneous dual active sites. *Angew. Chem. Int. Ed.* **59**, 16459-16464 (2020).
4. Ma W. et al. Electrocatalytic reduction of $CO_2$ to ethylene and ethanol through hydrogen-assisted C–C coupling over fluorine-modified copper. *Nat. Catal.* **3**, 478-487 (2020).
5. Yang P. P. et al. Protecting copper oxidation state via intermediate confinement for selective $CO_2$ electroreduction to C2+ fuels. *J. Am. Chem. Soc.* **142**, 6400-6408 (2020).
6. Hoang T. T. H. et al. Nanoporous copper-silver alloys by additive-controlled electrodeposition for the selective electroreduction of $CO_2$ to ethylene and ethanol. *J. Am. Chem. Soc.* **140**, 5791-5797 (2018).
7. Li F. et al. Cooperative $CO_2$-to-ethanol conversion via enriched intermediates at molecule–metal catalyst interfaces. *Nat. Catal.* **3**, 75-82 (2019).
8. Wu Z. Z. et al. Identification of Cu(100)/Cu(111) interfaces as superior active sites for CO dimerization during $CO_2$ electroreduction. *J. Am. Chem. Soc.* **144**, 259-269 (2022).
9. Niu Z. Z. et al. Hierarchical copper with inherent hydrophobicity mitigates electrode flooding for high-rate $CO_2$ electroreduction to multicarbon products. *J. Am. Chem. Soc.* **143**, 8011-8021 (2021).
10. Zhang X. et al. Selective and high current $CO_2$ electro-reduction to multicarbon products in near-neutral KCl electrolytes. *J. Am. Chem. Soc.* **143**, 3245-3255 (2021).
11. Xu B. H. et al. Highly efficient electrocatalytic $CO_2$ reduction to $C_{2+}$ products on a poly(ionic liquid)-based Cu(0)-Cu(I) tandem catalyst. *Angew. Chem. Int. Ed.* **61**, e202110657 (2021).
12. Liu C. et al. Nanoconfinement engineering over hollow multi-shell structured copper towards efficient electrocatalytical C-C coupling. *Angew. Chem. Int. Ed.* **61**, e202113498 (2021).
13. Yang B. et al. Accelerating $CO_2$ electroreduction to multicarbon products via synergistic electric-thermal field on copper nanoneedles. *J. Am. Chem. Soc.* **144**, 3039-3049 (2022).
14. Zhuang T.-T. et al. Steering post-C–C coupling selectivity enables high efficiency electroreduction of carbon dioxide to multi-carbon alcohols. *Nat. Catal.* **1**, 421-428 (2018).
15. Luo M. et al. Hydroxide promotes carbon dioxide electroreduction to ethanol on copper via tuning of adsorbed hydrogen. *Nat. Commun.* **10**, 5814 (2019).
16. Kim T. et al. A scalable method for preparing Cu electrocatalysts that convert $CO_2$ into $C_{2+}$ products. *Nat. Commun.* **11**, 3622 (2020).
17. Dinh C.-T. et al. $CO_2$ electroreduction to ethylene via hydroxide-mediated copper catalysis at an abrupt interface. *Science* **360**, 783-787 (2018).
18. Chen X. et al. Electrochemical $CO_2$-to-ethylene conversion on polyamine-incorporated Cu electrodes. *Nat. Catal.* **4**, 20-27 (2020).
